# Identification of Low-Voltage Areas: A Unipolar, Bipolar, and Omnipolar Perspective

**DOI:** 10.1161/CIRCEP.121.009912

**Published:** 2021-06-18

**Authors:** Mathijs S. van Schie, Rohit K. Kharbanda, Charlotte A. Houck, Eva A.H. Lanters, Yannick J.H.J. Taverne, Ad J.J.C. Bogers, Natasja M.S. de Groot

**Affiliations:** 1 Department of Cardiology (M.S.v.S., R.K.K., C.A.H., E.A.H.L., N.M.S.d.G.), Erasmus Medical Center, Rotterdam, the Netherlands.; 2 Department of Cardiothoracic Surgery (R.K.K., C.A.H., Y.J.H.J.T., A.J.J.C.B.), Erasmus Medical Center, Rotterdam, the Netherlands.

**Keywords:** atrial fibrillation, congenital heart disease, electrodes, electrophysiology, epicardial mapping, heart diseases

## Abstract

Low-voltage areas (LVAs) are commonly considered surrogate markers for an arrhythmogenic substrate underlying tachyarrhythmias. It remains challenging to define a proper threshold to classify LVA, and it is unknown whether unipolar, bipolar, and the recently introduced omnipolar voltage mapping techniques are complementary or contradictory in classifying LVAs. Therefore, this study examined similarities and dissimilarities in unipolar, bipolar, and omnipolar voltage mapping and explored the relation between various types of voltages and conduction velocity (CV).

**Methods:**

Intraoperative epicardial mapping (interelectrode distance 2 mm, ±1900 sites) was performed during sinus rhythm in 21 patients (48±13 years, 9 male) with atrial volume overload. Cliques of 4 electrodes (2×2 mm) were used to calculate the maximal unipolar, bipolar, and omnipolar voltages and mean CV. Areas with maximal bipolar or omnipolar clique voltage ≤0.5 mV were defined as LVA.

**Results:**

The maximal unipolar clique voltage was not only larger than maximal bipolar clique voltage but also larger than maximal omnipolar clique voltage (7.08 [4.22–10.59] mV versus 5.27 [2.39–9.56] mV and 5.77 [2.58–10.52] mV, respectively, *P*<0.001). In addition, the largest bipolar clique voltage was on average 1.66 (range: 1.0–59.0) times larger to the corresponding perpendicular bipolar voltage pair. LVAs identified by a bipolar or omnipolar threshold corresponded to a broad spectrum of unipolar voltages and, although CV was generally decreased, still high CVs and large unipolar voltages were found in these LVAs.

**Conclusions:**

In patients with atrial volume overload, there were considerable discrepancies in the different types of LVAs. Additionally, the identification of LVAs was hampered by considerable directional differences in bipolar voltages. Even using directional independent omnipolar voltage to identify LVAs, high CVs and large unipolar voltages are present within these areas. Therefore, a combination of low unipolar and low omnipolar voltage may be more indicative of true LVAs.

**WHAT IS KNOWN?**

- Low-voltage areas are commonly considered surrogate markers for arrhythmogenic atrial tissue containing areas of slow conduction, thereby serving as potential target sites for ablation therapy of atrial tachyarrhythmias.
- Voltage mapping considerably depends on the use of either unipolar, bipolar, or omnipolar electrograms, each having its own advantages and disadvantages. However, it remains unknown whether omnipolar voltages are complementary or contradictory to unipolar and bipolar voltages in identifying low-voltage areas.

**WHAT THE STUDY ADDS?**

- There are considerable directional differences in bipolar voltages; >20% of the largest bipolar voltage differed even >50% from the corresponding perpendicular bipolar voltages, having a major impact on identification of low-voltage areas.
- Using omnipolar voltage mapping, 15% of the bipolar low-voltage areas disappeared, although it also resulted in 2.6% additional low-voltage areas which had normal bipolar voltages. All low-voltage areas contained a large variety of unipolar voltages, and although conduction velocity was generally decreased, high conduction velocities and large unipolar voltages could still be found within these areas.
- In patients with interatrial left-to-right shunts, no predilection sites for low-voltage areas were found. However, all different types of voltage maps demonstrated interregional differences, and high interindividual unipolar voltage variations were found.

Low-voltage areas (LVAs) are commonly considered surrogate markers for arrhythmogenic atrial tissue containing areas of slow conduction, thereby serving as potential target sites for ablation therapy of atrial tachyarrhythmias, including intraatrial reentrant tachycardias and focal atrial tachycardias.^1–3^ However, whether LVAs also play a role in the pathogenesis of atrial fibrillation in patients with congenital heart disease remains unknown. Voltage mapping considerably depends on the use of either unipolar or bipolar electrograms, each having its own advantages and disadvantages.^1,4^

As unipolar electrograms comprise a larger region of myocardial electrical activity, bipolar recordings are mainly used to detect scar tissue areas as it represents more local information. Although ablation of bipolar LVAs has shown a possible benefit in certain patient populations, the efficacy of such bipolar voltage-guided ablation strategies remains controversial.^5–8^ This can only partly be explained by the complexity of bipolar electrograms and the directional sensitivity on the potential voltage, which decreases when wavefront propagation is perpendicular to the recording electrodes. To overcome the directional sensitivity, a so-called omnipolar mapping technique has been recently developed which mathematically extracts maximal bipolar voltage from a collection of electrograms, independently of wavefront propagation direction.^9–11^ However, it has been suggested that unipolar voltage mapping is preferred to identify intramural arrhythmogenic substrate.^1^ It is for these reasons that unipolar and bipolar voltage mapping are increasingly combined to provide additional information on the underlying tissue.^12–17^ Still, it remains very challenging to define a proper threshold to identify LVAs, and it also remains unknown whether unipolar, bipolar, and omnipolar voltages are complementary or contradictory on identifying LVAs at a high-resolution scale. Therefore, we performed high-density epicardial mapping in patients with atrial volume overload to (1) examine similarities and dissimilarities in unipolar, bipolar, and omnipolar voltage distribution and (2) explore the relation between various types of voltages and conduction velocity (CV) in identification of LVA.

## Methods

The data that support the findings of this study are available from the corresponding author upon reasonable request.

### Study Population

The study population consisted of 21 adult patients with atrial volume overload due to an interatrial left-to-right shunt undergoing surgical correction in the Erasmus Medical Center Rotterdam. This study was approved by the institutional medical ethical committee (MEC2010-054/MEC2014-393).^18,19^ Written informed consent was obtained from all patients. Patient characteristics were obtained from the patient’s medical record.

### Epicardial Mapping Procedure

Epicardial high-resolution mapping was performed before commencement to extracorporeal circulation, as previously described in detail.^20–22^ A temporal bipolar epicardial pacemaker wire attached to the right atrium (RA) free wall served as a reference electrode. A steel wire fixed to subcutaneous tissue of the thoracic cavity was used as an indifferent electrode. Epicardial mapping was performed with a 128-electrode array or 192-electrode array (GS Swiss PCB AG, Küssnacht, Switzerland; interelectrode distance 2.0 mm, both vertically and horizontally; electrode diameter 0.45 mm; array surface 14×30 mm and 14×46 mm).^22^ Mapping was conducted by shifting the electrode array along imaginary lines with a fixed anatomic orientation, following a predefined mapping scheme, covering the entire epicardial surface of the RA, Bachmann bundle (BB), pulmonary vein area, and left atrium (LA).^18^ Omission of areas was avoided at the expense of possible small overlap between adjacent mapping sites. The RA was mapped from the cavotricuspid isthmus, shifting perpendicular to the caval veins towards the RA appendage. The pulmonary vein area was mapped from the sinus transversus fold along the borders of the right and left pulmonary veins down towards the atrioventricular groove. The left atrioventricular groove was mapped from the lower border of the left inferior pulmonary vein towards the LA appendage. BB was mapped from the tip of the LA appendage across the roof of the LA, behind the aorta towards the superior cavoatrial junction.

**Figure 1.**
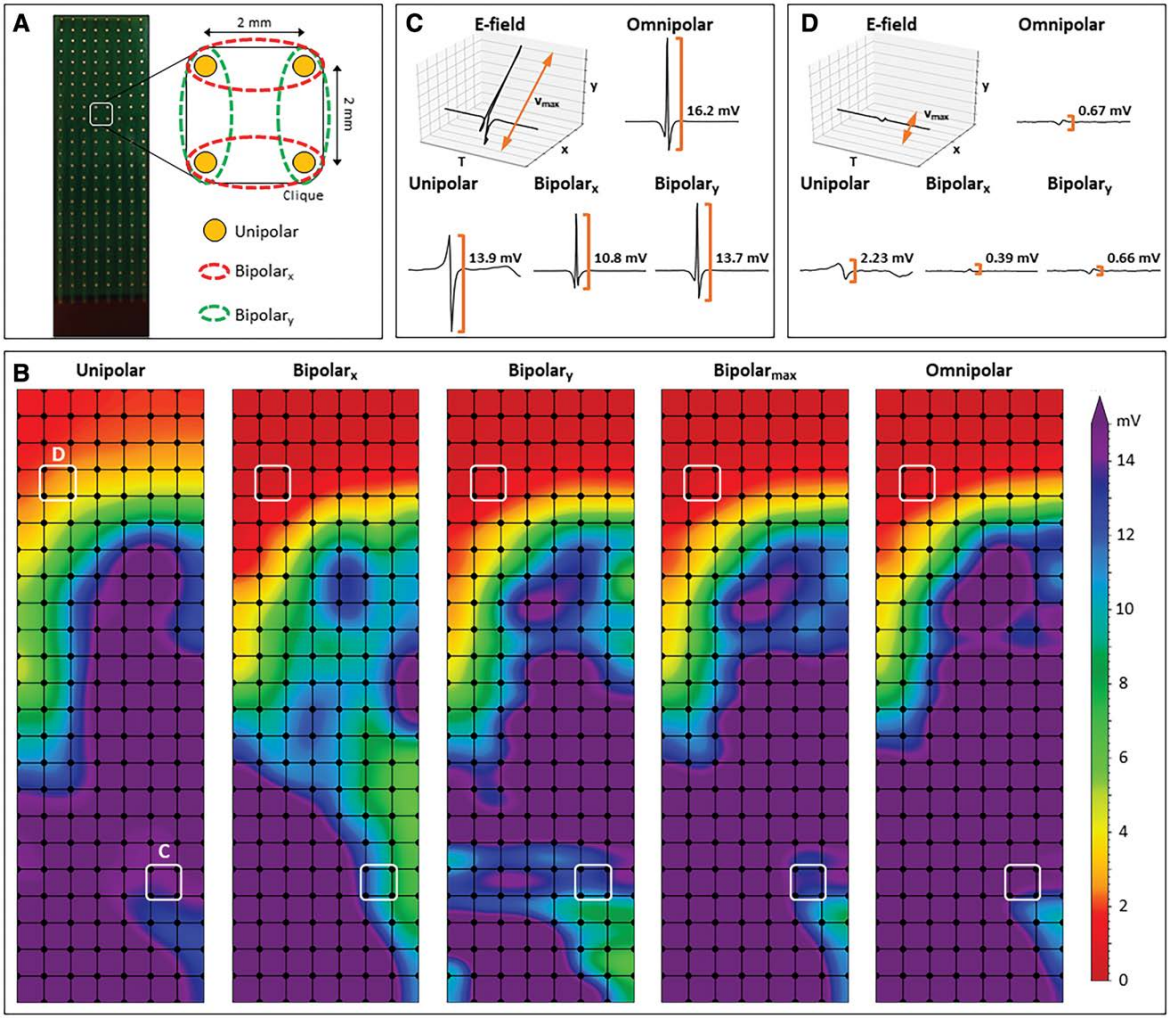
**Construction of unipolar, bipolar, and omnipolar voltages in 2x2 mm cliques. A**, A high-density electrode array consisting of 192 unipolar electrodes (2 mm interelectrode distance) was used to map the epicardial surface of the atria during open-chest surgery. For each square area, enclosed by 4 electrodes, 4 unipolar electrograms (EGMs), and matched bipolar and omnipolar EGMs were derived from 2 electrode orientations (along the vertical *y* axis [green] and horizontal *x* axis [red]) as indicated by the dotted lines. **B**, Peak-to-peak voltages of corresponding EGMs are used to create different voltage maps. The electrode orientation dependence of bipolar mapping is clearly visualized by the considerable differences in voltages between the bipolar-*x* and bipolar-*y* voltage maps. Bipolar-max voltage map illustrates the maximal bipolar voltage in both horizontal and vertical orientations within one clique. **C** and **D**, Examples of a unipolar, horizontal bipolar-*x*, vertical bipolar-*y*, and omnipolar EGM. The 2 bipolar EGMs differed considerably, illustrating the electrode orientation dependence of bipolar mapping. Omnipolar mapping provides electrode orientation-independent voltages that are larger (**C**) and similar (**D**) to the bipolar with the largest measurable peak-to-peak voltage, in both cases the vertical bipolar-*y* EGMs.

Five seconds of stable sinus rhythm were recorded from every mapping site, including a surface ECG lead, a calibration signal of 2 mV and 1000 ms, a bipolar reference electrogram and all unipolar epicardial electrograms. Data were stored on a hard disk after amplification (gain 1000), filtering (bandwidth 0.5–400 Hz), sampling (1 kHz), and analog to digital conversion (16 bits). Bipolar electrograms were created by subtracting two neighboring unipolar electrograms in horizontal (bipolar-*x*) and vertical direction (bipolar-*y*) and subsequently filtered (bandwidth 30–400 Hz) as demonstrated in Figure 1A.

### Omnipolar Voltage Mapping

Omnipolar electrograms were created from the bipolar electrograms using a technique described by Deno et al.^10^ Within a square area defined by 4 adjacent electrodes (a clique), omnipolar electrograms were used to mathematically obtain bipolar electrograms in any direction without physically rotating the sensing electrodes of the bipolar pair. As demonstrated in Figure 1B, within a clique, a 2-dimensional voltage vector 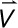 is derived from an electric field of a passing activation wavefront from which the maximal extend of 2 orthogonal bipolar electrograms is calculated over the interval (*T* )^9^:


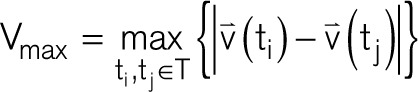


V_max_ corresponds to the peak-to-peak amplitude of a bipolar voltage signal obtained along the unit vector direction 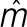 where t_i_ and t_j_ are now the times associated with V_max_ in which t_i_ > t_j_:


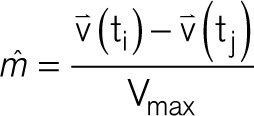


V_max_ provides an objective measure of the largest possible bipolar electrogram within a clique without the ambiguity of electrode orientation and is used to describe omnipolar electrogram voltages.

### Data Analysis

Unipolar, bipolar, and omnipolar electrograms were semi-automatically analyzed using custom-made software. The steepest negative slope of a unipolar atrial potential was marked as the local activation time, providing that the amplitude of the deflection was at least 2× the signal-to-noise ratio of the unipolar electrogram. Double and fractionated potentials were defined as potentials with respectively 2 and ≥3 deflections. All annotations were manually checked with a consensus of 2 investigators. CV was computed from local activation times using discrete velocity vectors as previously described.^23^ Signal voltage was defined as the peak-to-peak amplitude of the steepest deflection (unipolar) or highest peak (bipolar and omnipolar), as demonstrated in Figure 1B. As omnipolar electrograms can only be derived in square areas, unipolar and bipolar potentials were correlated to each other in areas of 2×2 mm—a clique—which contain 4 unipolar electrograms, the corresponding bipolar-*x*/*y* electrograms and the omnipolar electrogram (Figure 1A). Subsequently, the maximal potential voltage of the unipolar electrograms, maximal potential voltage of the bipolar-*x*/*y* electrograms and omnipolar electrogram pertaining to that area were computed, resulting in 3 values (V_uni,max_, V_bi,max_, and V_omni,max_). In addition, the mean of the 4 CV estimates derived from the 4 unipolar local activation times was used as indication of the CV through the 2×2 mm area. As a bipolar voltage cutoff of ≤0.5 mV is most frequently used in daily clinical practice to identify LVAs, we also used this value as the golden standard to identify low-voltage cliques.^24^ Areas corresponding to a mean CV of 0 cm/s were excluded to avoid the inclusion of far-field potentials.

### Statistical Analysis

Normally distributed data are expressed as mean±SD, whereas skewed data are expressed as median (25th–75th percentile). Clinical characteristics were compared using the Student *t* test or Mann-Whitney *U* test when appropriate. Categorical data are expressed as numbers (percentages) and analyzed with a χ^2^ or Fisher exact test. Paired voltage data was analyzed between patients using the Wilcoxon signed-rank test. A *P*<0.05 was considered statistically significant. A Bonferroni correction was applied when appropriate.

## Results

### Study Population

Clinical characteristics of the study population (N=21, age 48±13 years, 9 male (43%)) are summarized in Table 1. Most patients had an atrial septal defect type II (N=12, 57%). The other patients had a sinus venosus defect with partial abnormal pulmonary venous return (N=7) and atrial septal defect type I (N=1) and isolated partial abnormal pulmonary venous return (N=1). RA and LA dilatation were, respectively, present in 19 (90%) and 5 (24%) patients. Patients had no history of atrial arrhythmias.

**Table 1.**
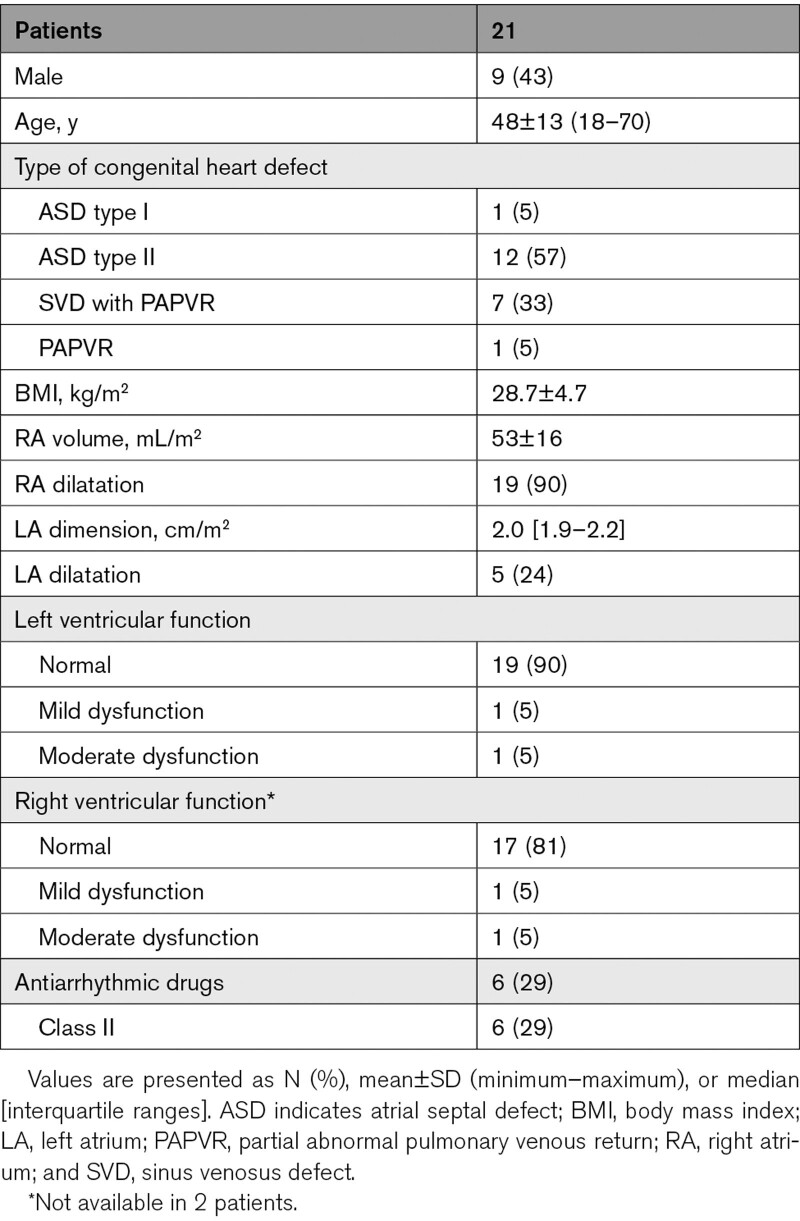
Baseline Characteristics

### Unipolar, Bipolar, and Omnipolar Voltage Maps

In the entire study population, a total of 193 mapping locations resulted in 175 667 unipolar and 306 685 bipolar recordings from which 146 015 cliques were created. Within the 2×2 mm areas, there were considerable directional differences in bipolar voltages. The largest bipolar voltage was on average 1.66 (ranging from 1.0 to 59.0) times larger than the corresponding perpendicular bipolar voltages.

Differences in voltage maps constructed by using unipolar and corresponding bipolar-*x*, bipolar-*y*, or omnipolar electrograms are illustrated in Figure 1B. Figure 1C shows the electrograms derived from the highlighted area at the bottom of the mapping array. The largest unipolar electrogram had an amplitude of 13.9 mV, while its corresponding bipolar-*x* and bipolar-*y* electrograms had an amplitude of, respectively. 10.8 and 13.7 mV. The amplitude of the corresponding omnipolar electrogram was larger compared with the largest bipolar-*x*/*y* electrogram (16.2 mV). Figure 1D shows the electrograms derived from the highlighted area at the top of the mapping array containing smaller voltages. Likewise, the largest unipolar electrogram (2.23 mV) was much larger than the corresponding bipolar-*x* (0.39 mV) and bipolar-*y* electrograms (0.66 mV). However, it now critically depends on the bipolar electrode orientation whether this clique was identified as LVA as only the bipolar-*y* electrogram was >0.5 mV. Furthermore, the corresponding omnipolar electrograms only resulted in an amplitude slightly larger than the largest bipolar-*x*/*y* electrogram (0.67 mV). As a result, there are differences between corresponding unipolar, bipolar, and omnipolar voltage maps, as demonstrated in Figure 1B.

**Figure 2.**
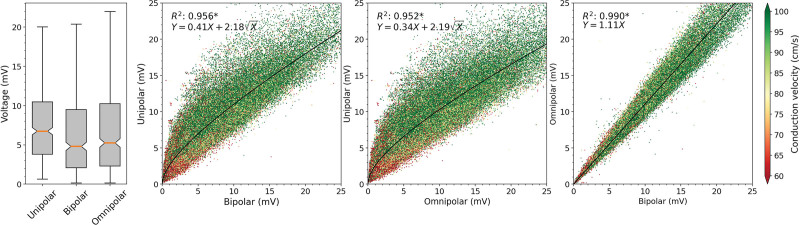
**Relation between unipolar, bipolar, and omnipolar voltages and CV.** Quantitative analysis of unipolar clique voltage (V_uni,max_), bipolar clique voltage (V_bi,max_), and omnipolar clique voltage (V_omni,max_) distributions (left) and the similarity of V_uni,max_, V_bi,max_, and V_omni,max_ voltages (other). The conduction velocity (CV) is color coded, ranging from 60 to 100 cm/s, visualized in which green represents high CV and red low CV. A black line indicates the ordinary least squares prediction. Statistical significance is indicated by an asterisk (*P*<0.001).

The left of Figure 2 demonstrates the distribution of V_uni,max_, V_bi,max_, and V_omni,max_ from all cliques obtained from all patients. V_uni,max_ was larger than both V_bi,max_ and V_omni,max_ (7.08 [4.22–10.59] mV versus 5.27 [2.39–9.56] mV and 5.77 [2.58–10.52] mV, respectively, *P*<0.001 for each). In addition, V_omni,max_ was larger than V_bi,max_ (5.77 [2.58–10.52] mV versus 5.27 [2.39–9.56] mV, *P*<0.001).

### Relationship Between Unipolar, Bipolar, and Omnipolar Voltages and CV

The right of Figure 2 demonstrates the relationship between V_uni,max_, V_bi,max_, and V_omni,max_. The mean CV of each clique was 92.0 (73.2–109.1) cm/s and is indicated by color-coded scatters; larger voltages (both V_uni,max_ and V_bi,max_) are associated with higher CVs. Double and fractionated potentials were present in 36.9% of the cliques. In only 24.7% of the cliques, bipolar voltages were larger than unipolar voltages. As a consequence, there was a strong inversely quadratic relation with linear component (*R*^2^=0.956; 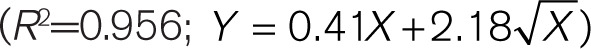 between V_uni,max_ and V_bi,max_. Similar results were obtained by comparing V_uni,max_ and V_omni,max_ (*R*^2^=0.952; 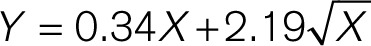 Furthermore, there was a strong, positive linear correlation (*R*^2^=0.990, *Y*=1.11*X*) between V_bi,max_ and V_omni,max_. When all V_bi,max_ are subdivided into 3 groups (<0.5, 0.5–1.0, and >1.5 mV) and compared with the corresponding V_omni,max_, there was an increasing influence of V_omni,max_ on the different V_bi,max_ groups (<0.5 mV: 4.4%; 0.5–1.0 mV: 5.0%; and >1.5 mV: 8.8%; all *P*<0.001). However, in terms of absolute values, the added effect of V_omni,max_ on lower voltages was only minimal (±0.02 mV).

### Characteristics of LVAs

In our data, the fifth percentile of the relative frequency V_bi,max_ histogram was 0.55 mV, which is comparable to the voltage cutoff value of ≤0.5 mV which is most frequently used in daily clinical practice to identify LVAs. We, therefore, also used this value to identify low-voltage cliques.

**Figure 3.**
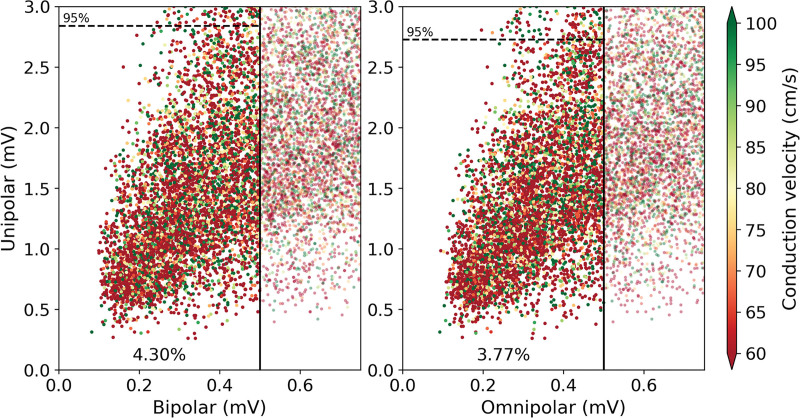
**Characteristics of LVAs.** Visualization of unipolar clique voltage (V_uni,max_) in low-voltage areas (LVAs; left of black solid line) identified using the golden standard threshold of 0.5 mV applied on bipolar clique voltage (V_bi,max_; left) and omnipolar clique voltage (V_omni,max_; right). Conduction velocity (CV) is color-coded visualized in which green represents high CV and red low CV. The dashed line indicates the 95th percentile of V_uni,max_ within the corresponding LVA. A 4.30% of V_bi,max_ was classified as LVA and 3.77% of V_omni,max_.

Application of this threshold on V_bi,max_ and V_omni,max_ is demonstrated in Figure 3. As shown in the left, respectively, 4.30% of V_bi,max_ and 3.77% of V_omni,max_ were classified as LVA; the corresponding V_uni,max_ and CV of these cliques are listed in Table 2. For both recording techniques, V_uni,max_ and CV was lower in LVAs, and double and fractionated potentials were more often recorded from these areas compared with normal areas (*P*<0.001 for all). Using only either the bipolar-*x* or -*y* values of all cliques, respectively, 37% and 21% additional cliques were classified as LVA.

**Table 2.**
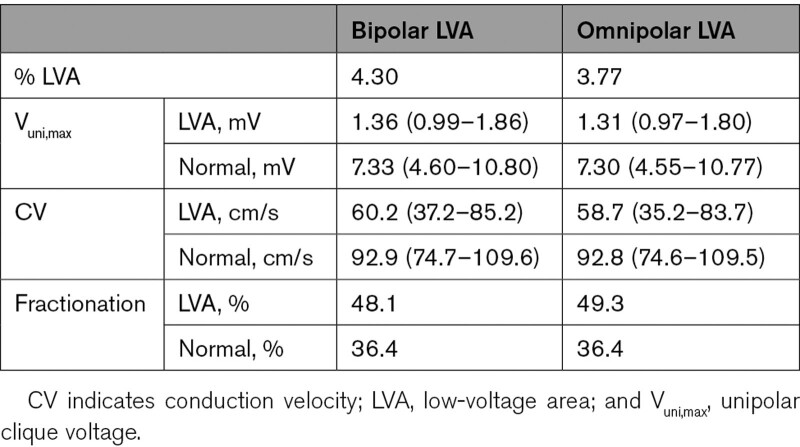
Characteristics of LVA (N=146 015)

When the threshold of 0.5 mV was applied to V_omni,max_ clique values, 14.6% of the bipolar LVA cliques were now identified as normal area. Although the majority of V_bi,max_ in these areas were in a relatively small range, a great variety of V_uni,max_ and CV was found (V_bi,max_ of 0.46 [0.41–0.48] mV ranging from 0.17 to 0.499 mV, corresponding V_uni,max_ of 1.77 [1.31–2.27] mV and CV of 69.1 [47.0–92.6] cm/s). However, 2.6% of the omnipolar LVA cliques (0.1% of all cliques) had normal V_bi,max_ values and were so falsely identified as LVA. However, this only accounted for cliques which were already very close to 0.5 mV; V_omni,max_ in these LVAs was 0.49 (0.47–0.49) mV against the corresponding V_bi,max_ of 0.52 (0.51–0.53) mV.

### Does a Unipolar Low-Voltage Threshold Exist?

As demonstrated in Figure 2, low V_bi,max_ and V_omni,max_ are related to V_uni,max_ by, in particular, the inversely quadratic component of the relationships. Therefore, a small increase in either V_bi,max_ or V_omni,max_ will result in a relatively large increase in V_uni,max_. This relation can be used to determine a unipolar threshold to identify the LVAs using only V_uni,max_. To detect the golden standard bipolar LVAs of ≤0.5 mV with a precision of at least 90%, a unipolar threshold of 0.53 mV should be used. Vice versa, by using this unipolar threshold, only 1.8% of the bipolar LVAs was classified as true positive. However, applying the golden standard threshold of ≤0.5 mV for V_omni,max_, a unipolar threshold of 0.48 mV should be used. Using this unipolar threshold only 1.1% of the omnipolar LVAs could be identified as true positive. Hence, using solely V_uni,max_, only a limited number of these golden standard LVAs could be correctly identified, and therefore, usage of V_uni,max_ alone is not suitable in identifying these LVAs.

### Patient Voltage Fingerprints

**Figure 4.**
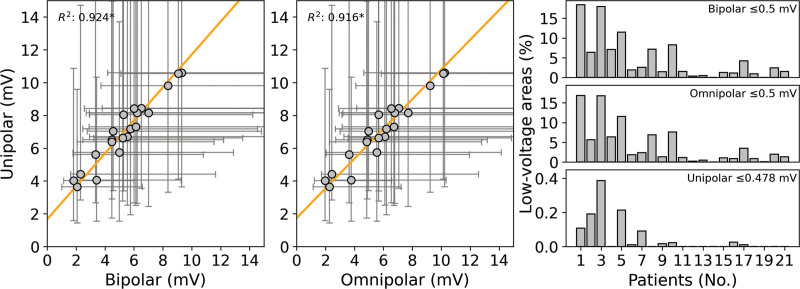
**Patient voltage fingerprints.** Relation of the patient voltage distributions (left and center). Each dot represents the median value of the corresponding individual unipolar and bipolar voltage distribution (**left**) or unipolar and omnipolar voltage distributions (**center**). The error bars represent the 25th and 75th quartiles of each distribution. The relationship is illustrated by the orange line. Statistical significance of the relationship is indicated by an asterisk (*P*<0.01). The **right** show the amount of low-voltage areas (LVAs; **top**: bipolar ≤0.5 mV; **middle**: omnipolar ≤0.5 mV; and **bottom**: unipolar ≤0.478 mV) in each patient separately ranked by the median bipolar voltage.

Next, all types of clique voltages were collected for each individual patient. As illustrated in Figure 4, there was a strong linear relationship between median V_uni,max_ and corresponding median V_bi,max_ and V_omni,max_ for every patient separately (*R*^2^=0.924, *P*<0.01 and *R*^2^=0.916, *P*<0.01, respectively). Voltage distribution of V_uni,max_, V_bi,max_, and V_omni,max_ varied considerably between various patients, as listed in Table 2. The largest median voltages were found in V_uni,max_ (7.03 [5.74–8.15] mV), followed by V_omni,max_ (5.68 [4.85–6.80] mV) and V_bi,max_ (5.27 [4.47–6.22] mV, *P*<0.001 for each).

Applying the golden standard threshold of 0.5 mV on V_bi,max_, LVAs were present in all patients (1.9% [1.1%–7.1%] ranging from 0.01% to 18.5%), while applying this threshold on V_omni,max_, LVAs were present in only 19 out of 21 patients (1.8% [0.9%–4.9%], ranging from 0.2% to 16.8%). The amount of LVAs was smaller when using V_omni,max_ compared with V_bi,max_ (*P*<0.001). As demonstrated in the left of Figure 4, patients with higher median V_bi,max_ had smaller amounts of LVAs compared with patients with lower median V_bi,max_. When applying the unipolar threshold of ≤0.48 mV on V_uni,max_, LVAs were only present in patients who also have a large amount of bipolar and omnipolar LVAs.

Next, all parameters were subdivided according to the corresponding atrial recording regions (RA, BB, pulmonary vein area, and LA) and are demonstrated in Table 3. V_uni,max_ was larger at LA compared with all other atrial regions (9.65 [7.16–10.73] mV versus RA: 6.21 [5.30–7.67] mV, BB: 6.81 [5.31–9.15] mV, and pulmonary vein area: 6.86 [3.64–8.32] mV, all *P*<0.0083), and V_bi,max_ and V_omni,max_ were larger at LA compared with RA and BB (V_bi,max_ LA: 6.88 [5.03–9.87] mV versus RA: 4.91 [3.93–6.57] mV and BB: 4.30 [2.54–6.11] mV; V_omni,max_ LA: 7.55 [5.43–11.11] mV versus RA: 5.55 [4.23–7.28] mV and BB: 4.73 [2.69–6.37] mV, all *P*<0.0083). There were no regional differences in CV, amount of bipolar LVAs and omnipolar LVAs.

**Table 3.**
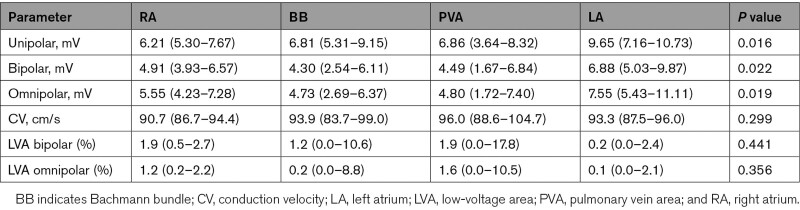
Regional Voltage Mapping Results (N=21)

## Discussion

### Key Findings

High-resolution voltage mapping in patients with congenital heart disease demonstrated that within an area of 2×2 mm, omnipolar voltages were larger than maximal bipolar voltages but smaller than maximal unipolar voltages. There were considerable directional differences in bipolar voltages; >20% of the largest bipolar voltage differed even >50% from the corresponding perpendicular bipolar voltages. These differences have a major impact on identification of LVAs. Using omnipolar voltages, 15% of the bipolar LVAs were not identified, although it also resulted in 2.6% additional LVAs which had normal bipolar voltages. All LVAs contained a large variety of unipolar voltages, and although CV was generally decreased, high CVs and large unipolar voltages could still be found within these areas. Due to high interindividual unipolar voltage variations within LVAs, no clear unipolar threshold corresponded with correct identification of LVAs in patients with interatrial left-to-right shunts. Although all different types of voltage maps demonstrated interregional differences, no predilection sites for LVAs were found.

### Voltage Mapping

In current clinical practice, atrial arrhythmogenic areas are identified using bipolar voltage mapping or by visualization of fibrotic areas using imaging techniques such as magnetic resonance imaging. Although there is still limited theoretical understanding of the determinants of bipolar electrograms, their amplitude has become the backbone of clinical substrate mapping approaches.^25^ Areas with low bipolar voltages are regarded as indicators of arrhythmogenic tissue and used as target sites for ablation therapy.^2^ However, bipolar voltage is not only affected by the underlying myocardial tissue but also by tissue proximity, cycle length, CV, fiber orientation, and curvature. Additionally, characteristics of the recording electrode such as the angle of the electrodes on tissue, interelectrode distance and electrode size may influence bipolar electrogram voltages as well.^1,25–28^

Although frequently debated, a recent study has shown that in simulated and clinical data, the amplitude of bipolar electrograms changes from a maximum value parallel to the propagation direction to 0 mV perpendicular to the propagation direction. This direction dependency may account for up to a 49% difference in bipolar voltage particularly during sinus rhythm.^4,9,26^ In our study, >20% of the largest bipolar voltage differed even >50% than the corresponding perpendicular bipolar voltages. Therefore, bipolar electrograms at one orientation could erroneously indicate that certain areas are diseased but not if examined at another orientation. In our study, 3.7% of the investigated areas could be identified as LVA if only one electrode orientation was taken into account. This would have resulted in a LVA overestimation of 17.6%. Therefore, by using solely bipolar electrograms, (non)arrhythmogenic areas can possibly be misclassified.

To overcome the directional dependency of bipolar voltage mapping, omnipolar electrograms have been introduced to improve substrate mapping by providing wavefront orientation-independent measurements revealing the highest possible bipolar voltage.^9,11^ However, the advantage of omnipolar mapping on low-amplitude voltages is relatively low due to its linear relationship with the corresponding bipolar voltages. The same implies for unipolar voltages that are also unaffected by the electrode orientation and electrode distances, although unipolar electrograms are more susceptible to noise and (ventricular) far field. Moreover, it is assumed that unipolar electrograms have a deeper field of view in the myocardium, which improves detection of intramural and endocardial or epicardial arrhythmogenic tissue.^1^ This can also explain the large range of unipolar voltages compared with the bipolar voltages within the cliques.

### Interrelationship Between Voltage and CV

It is generally assumed that bipolar voltage is affected by several factors, including CV.^1^ Multiple studies showed clear associations between bipolar voltage and CV, in which it was predominantly stated that areas of low bipolar voltage are associated with low CV.^26,29^ Due to the slowed conduction in these areas, neighboring unipolar electrograms may overlap resulting in a low-amplitude bipolar electrogram. This is similar to constructing a bipolar electrogram perpendicular to the propagation direction. In a study of Itoh et al^30^ a logarithmic relationship was found between RA CV and local bipolar voltage during atypical atrial flutter. Although in our study, larger voltages were associated with higher CVs, we could not find a clear relationship between CV and bipolar voltage as there was too much variation in CV across all recorded bipolar voltages.

Unipolar voltage has also previously been linked to CV. Fast conduction along the longitudinal axis of the atrial fibers is characterized by large unipolar voltages, whereas in areas of slowed conduction unipolar potentials have low amplitude.^31^ In addition, loss of S-wave amplitude in patients with paroxysmal atrial fibrillation and decrease of peak negative voltage during atrial flutter have been previously correlated to a decrease of CV.^32,33^ This is in accordance with our study, in which lower CVs were predominantly found in areas with lower unipolar voltages.

### Ablation Targeting LVAs

Multiple ablation strategies of atrial and ventricular tachyarrhythmias target diseased myocardium identified by bipolar voltage, which is associated with structurally remodeled areas with local slowing of conduction. Abnormal LVAs in the atria are usually identified with voltage cutoff values in sinus rhythm of ≤0.5 mV and scar ≤0.05 mV as it is then indistinguishable from noise.^25,34^ However, there is still much debate on which thresholds to use and when classifying tissue as diseased, healthy, or as an intermediate zone that does not contain substantial remodeled areas but also not only healthy tissue.^34^ In addition, Soejima et al^35^ have shown that there are still surviving excitable fibers within LVAs, which are important pathways within reentry circuits underlying ventricular tachycardias. From our data, it is clear that there is no single straightforward method to identify arrhythmogenic areas, but different grades can be observed.^34^

It becomes even more difficult as directional sensitivity of bipolar electrograms limit the accuracy of these approaches by causing underestimation of bipolar voltage, especially when using narrowly spaced electrodes and in regions with normal CV.^26^ Although unipolar electrograms are not affected by directional sensitivity, substrate assessment by solely unipolar voltage is also limited.^36^ We found that even in omnipolar LVAs there are still high unipolar voltages and a large variety of CVs. The logarithmic relationship between RA CV and bipolar voltage found by Itoh et al^30^ also shows that for a relatively narrow interval of bipolar voltages a broad spectrum of CVs can be found. In this study, it was reported that all bipolar voltages ≤0.5 mV covered more than half of the CVs. This is comparable to our results, in which we also found a wide range of CVs within the LVAs. Furthermore, we also demonstrated that unipolar and bipolar voltages could vary considerably in LVAs. Hence, low bipolar voltages do not necessarily represent an arrhythmogenic substrate. For these reasons, fixed voltage thresholds are questionable.

Several studies also reported on the combination of unipolar and bipolar voltage mapping to characterize the atrial substrate.^15,37,38^ Although results were mixed, Chopra et al^37^ proposed that the mismatch between bipolar and unipolar LVAs represents zones of scar that extend deep to and beyond the endocardial abnormal voltage area. This is in line with the deeper field of view of unipolar electrograms. Furthermore, it has been suggested that the bipolar voltage threshold of 0.5 mV overestimates the size of dense scar and still harbors islets and channels of viable tissue, while a unipolar threshold of <1.0 mV showed no discernible or excitable tissue and represents electrically dense scar. LVAs containing both low omnipolar and low unipolar voltage could, therefore, be more indicative of ‘true’ arrhythmogenic tissue.

### LVAs and Congenital Heart Disease

Especially in patients with an interatrial shunt, significant interindividual variation in the spatial distribution of atrial conduction disorders exist even during sinus rhythm.^38–41^ In addition, lower voltages, both unipolar and bipolar, were found in this patient population as compared to patients without structural heart disease, although normal and scar tissue could not clearly be delineated.^17^ Larger LVAs in patients with congenital heart disease and complex atrial tachyarrhythmias are associated with worse acute and midterm clinical outcomes.^42^ As demonstrated by Houck et al,^39^ conduction disorders in patients with an interatrial shunt are most pronounced in the RA and BB. Furthermore, other studies showed the presence of functional conduction delay in the region of the crista terminalis in patients with (chronic) atrial overload, which are related to development of atrial tachyarrhythmias.^24,40,43^ In our study, we found a large interindividual and interregional variety of unipolar, bipolar, and omnipolar voltages.

Although conduction disorders are frequently present in this patient population, LVAs were not identified in all patients. Specifically, LVAs were most often found at the RA, although the amount of LVAs did not differ between all atrial regions. The patients with LVAs, however, showed not only a large variety in the amount of LVAs but also corresponding unipolar voltages varied considerably. Also, although CV was generally decreased, still high CVs were found in these areas while LVAs are commonly considered surrogate markers for diseased atrial tissue with slowed conduction. Therefore, accurate identification of target sites for ablation therapy can be very challenging in this patient population.

### Study Limitations

This study focused on the comparison of the different voltage mapping methodologies and identification of LVAs without interventions. The next step will be to incorporate the results of this study with ablation targeting LVAs to determine whether the combination of low unipolar and low omnipolar voltage can improve ablation outcomes.

### Conclusions

In patients with congenital heart disease, there were considerable discrepancies in the different types of LVAs, which are particularly important as LVAs are considered surrogate markers for arrhythmogenic tissue. There were considerable directional differences in bipolar voltages hampering identification of LVAs. Even using directional independent omnipolar voltage to identify LVAs, high CVs and large unipolar voltages could still be found within these areas. In addition, the added value of omnipolar voltage in identifying LVAs is questionable as the amount of LVAs was only minimally decreased using this technique compared with maximal bipolar clique voltage. Given the various (often nonsubstrate related) factors affecting bipolar voltage, a combination of low omnipolar and low unipolar voltage may be more indicative of true LVAs rather than only one approach. Future studies are required to determine whether incorporation of unipolar voltage in these techniques to guide ablative therapy increases the ability to identify true LVAs and thus diseased atrial tissue.

## Acknowledgments

We kindly thank J.A. Bekkers, MD, PhD; C. Kik, MD; W.J. van Leeuwen, MD; F.B.S. Oei, MD, PhD; P.C. van de Woestijne, MD; F.R.N. van Schaagen, MD; A. Yaksh, MD, PhD; C.P. Teuwen, MD, PhD; E.M.J.P. Mouws, MD, PhD; J.M.E. van der Does, MD, PhD; R. Starreveld, PhD; C.S. Serban, DVM; L.N. van Staveren, MD; A. Heida, MD; W.F.B. van der Does, MD; and M.C. Roos-Serote, PhD, for their contribution to this work.

## Sources of Funding

Prof de Groot, MD, PhD, is supported by funding grants from CVON-AFFIP (grant no. 914728), NWO-Vidi (grant no. 91717339), Biosense Webster USA (ICD 783454), and Medical Delta.

## Disclosures

None.
